# Novel T-ring-based probes for thermal ablation of tumors in different organs

**DOI:** 10.1038/s41598-025-28192-2

**Published:** 2025-12-05

**Authors:** Menna Asran, Mahmoud Farouk Selim, Amira S. Ashour

**Affiliations:** 1 Department of Electronics and Electrical Communications Engineering, Faculty of Engineering, Tanta University, Tanta, Egypt; 2 Faculty of Medicine, Tanta University, Tanta, Egypt

**Keywords:** Finite element method, Thermal ablation, Tumors, Single air slot probe, Double slot sleeved probe, Cancer, Engineering, Medical research, Oncology, Physics

## Abstract

Thermal ablation has arisen as a crucial procedure for managing several solid tumors, presenting a minimally invasive choice that induces tumor mortification through localized heating. However, tumors may locate in different anatomical areas/organs, including the liver, kidney, lung, and breast. Generally, thermal ablation techniques use specific probe design that matches the tumor site and size, highlighting a noteworthy research gap in the implementation of an effective versatile probe that ablates tumors of various organs. This inspired this study to design an innovative thermal ablation T-Ring-based probes, namely the single slot with 3 T-rings (3TSS) probe, and single slot sleeved with 10 T-rings (10TSSS) probe, that enthused by the traditional single air slot probe, and the double slot sleeved probe, respectively. The proposed probes can be used at different sites in the human body, namely liver, breast, kidney, and lung. The proposed probes optimize the dissipated power consumption during the ablation process. The optimal probe’s impedance is obtained by analyzing the dissipated power values with achieving the best S11 reflection coefficient values. The experimental results achieved -21.553 dB, and -24.816 dB reflection coefficients of the two new probes, 3TSS, and 10TSSS, respectively. In addition, the results validated the proposed finite element model of the proposed probe compared to a real ablation probe.

## Introduction

Conventional local ablative devices have been established for managing tumors using radio-frequency ablation, laser ablation, cryoablation, and ethanol ablation. Heat-based thermal therapy can be managed using electromagnetic fields at specific radio frequencies [1, 2], microwave frequencies [3, 4], laser frequencies, and ultrasound frequencies, to transfer heat through tissues that destroy tumors. It basically depends on increase the temperature above the normal physiological level of 40 degrees Celsius (°C) [5], thereby affecting the cellular functionality and the tissues. Raising tissue’s temperature to 41 °C improves the blood flow and the ions diffusion across cell membranes, increasing the cellular sensitivity to damage [6]. Raising the temperature within the range from 41 to 48 °C for 30 min-1 h leads to cell destruction [7]. Once the temperature exceeds 48 to 60 °C, necrosis and coagulation occur, while at 150 °C drying, evaporation, and charring of the tissues happen [8]. In biological tissues, the heat transfer process is affected by the heat production from metabolism, blood flow, thermal conduction/ convection, water evaporation, and phase change [9, 10]. The treatment methods primarily depend on the patient’s condition.

Currently, microwave technology is becoming increasingly popular in the treatment of tumors [11, 12] by raising the temperature of the tumor’s tissues through interstitial hyperthermia and thermal ablation [6]. In microwave ablation (MWA), a thin probe is inserted into the patient’s body through a skin puncture during anesthesia [6]. The entire ablation process takes several minutes depending on the tumor’s size, the total used power, and the probe’s operating frequency, and type. In the last decade, significant developments have been observed in the designs of different probes [5, 7, 13]. These advances include the evolution from early interstitial microwave antennas with limited heating control [14, 15] to dual-slot and multi-slot configurations capable of producing more uniform and spherical ablation zones [16–18], as well as optimized and conformal designs tailored for specific tumor geometries [19, 20]. Several types of probes have been developed to deliver high temperature to the surrounding tissues around the microwave probe for ablating the tumor. These probes include coaxial probes with single air slot [13], double air slots [21], and multi-slot air slots [5], floating sleeve [21], and helical probes [3]. Coaxial antennas can be used alone or arranged in an array to direct the electromagnetic radiation inside the tumor for realizing larger ablation area in a short time [7].

The single air slot coaxial probe is considered the milestone for any further designs, as well as the double air slot probe, which contains a floating sleeve. For example, the single slot with 3 T-rings probe relied on adding T-shaped rings around the axis of the single air slot probe to control the shape of the dissipated power around the probe. The performance of the probe is affected by changing the distance between the rings, the number of rings, and the distance between the rings and the air slot. Also, a single slot sleeved with 10 T-rings probe was developed by changing the location of the air slot, adding a floating sleeve, changing its length, and placing rings on it. Various probe configurations were evaluated in terms of the probe performance by simulating the different structures and their effects on the tissues [22], time, and cost associated with experiments [23].

A comparison was conducted across different probes in terms of the dissipated power in the tissues [24]. Healthy liver tissues containing a liver tumor were simulated using two developed probes exhibited improved characteristics regarding the distribution of power dissipation around the probe’s tip and axis. This, in turn, influenced the configuration of the ablated tissues surrounding the probe, including both tumor tissue and the adjacent healthy tissue, as well as the shape of the isothermal contours and the maximum achieved temperature by each probe under identical conditions. The configuration of the ablated tissue nearby the probe is primarily determined by the amount of heat transferred to the tissues [25]. The greater the power dissipated by the probe, the more heat is transferred to the tissue, resulting in faster ablation of the tissue. The reflection coefficient S11 of each probe was also measured [8]. The lower the reflection coefficient, the more efficiently the probe consumes power. The single slot sleeved with 10 T-rings probe demonstrated the highest performance, recording − 24.816 dB at a frequency of 2.45 GHz.

Therefore, the proposed is conducted using a single slot sleeve with a 10 T-rings probe, comparing four vital tissues: liver, breast, kidney, and lung, where tumors are present in each tissue [26].

The traditional probes, such as the single air slot probe, and the double slot sleeved probe, were characterized by the simplicity of the structure. However, they caused backward heat that leads to burning healthy tissues around the probe with power wasting. Accordingly, in this work, the backward heat as well as the power consumption was reduced to ensure maximum benefit from the two probes under study. Accordingly, the main contributions of this study are as follows:

1. Designing two novel thermal ablation probes to solve the limitations of the traditional single air slot probe, and the double slot sleeved probe, which are called: single slot with 3 T-rings (3TSS) probe, and single slot sleeved with 10 T-rings (10TSSS) probe.
2. Conducting a comparative study of the traditional and novel probes in terms of their shapes and internal structure. Also, the isotherm contours around each probe, the maximum temperature reached by the surrounding tissues, and the shape of the ablated part of healthy tissue and infected tissue were studied.
3. Achieving better shape of the dissipated power around the tip and axis of the proposed probes compared to the traditional one.
4. Studying the performance of the proposed probes with four different types of tissues: liver, breast, kidney, and lung. Different biological tissues were investigated in this study to analyze how their distinct dielectric and thermal properties influence the microwave energy absorption, heat transfer, and ablation behavior. Such comparison provides valuable insights into the performance of the proposed probes across various organs’ environments. Accordingly, it is concluded that while the antenna choice in practice depends mainly on ablation geometry and accessibility, understanding tissue-dependent interactions is essential for optimizing probe design and predicting clinical performance [27, 28].
5. Conducting a comparative, computational study between the two proposed probes: 3TSS and 10TSSS in terms of the power dissipated, reflection coefficient, isotherm contours, and the ablated tissues using the same power level.
6. Assessing the effectiveness of the proposed FEM-based simulation in examining the development of the probe structure and its impact on the tissues with a practical experiment involving porcine lung tissue using the same probe and under identical experimental conditions. A convergence between the results recorded from the practical experiment and those from the simulation was proved.

## Material and methods

A finite element method was used to simulate the design of the proposed 3TSS probe, and the10TSSS probe, as well as to identify the most efficient required power for ablating different tumor types and the exposure times under various conditions. The proposed optimal power produces heat through electromagnetic waves to ablate the tumor. The heat transfer occurs due to the radiation from electromagnetic waves generated by the source at a frequency of 2.45 GHz. In addition, the electromagnetic energy (EM) radiation is represented by Maxwell’s equations.

### Mathematical background and finite element method

For optimizing the proposed probe’s positioning and the required input power during the ablation process, the principle of heat transfer was applied showing the thermal field distribution during MWA. The electric field position was determine using numerical methods of computational EM wave, while the temperature field distribution was calculated using the bioheat transfer equation [13]. The propagation of the EM wave in a coaxial cable is mathematically expressed. The electric and magnetic fields associated with the time-varying transverse electromagnetic waves are generated. A microwave source propagating in the coaxial cable in the Z direction is expressed in two-dimensional symmetric cylindrical coordinates as follows [22, 30]:

1 $$\overline{\rm E} (r) = e_{r} \frac{{ce^{j(\omega t - kz)} }}{r},$$

2 $$\overline{\rm H} (r) = e_{\varphi } \frac{{ce^{j(\omega t - kz)} }}{{{\rm Z}_{die} r}},$$

3 $${\rm P}_{av} = \int\limits_{{r_{inner} }}^{{r_{outer} }} {{\mathrm{Re}} \left( {\frac{\rm E}{2} \times {\rm H}^{ * } } \right)} 2\pi rdr,$$

4 $${\rm P}_{av} = e_{z} \pi \frac{{c^{2} }}{{{\rm Z}_{die} }}\ln \left( {\frac{{r_{outer} }}{{r_{inner} }}} \right).$$

where the cylindrical coordinates *r*, $\varphi$, and *z* are centered on the coaxial axis of the cable. *z* is the direction of propagation, $\omega$ is the angular frequency, *k* is propagation constant, ${\rm P}_{av}$ is average power flow through the cable, ${\rm Z}_{die}$ is wave impedance in the cable dielectric, $r_{inner}$ and $r_{outer}$ are the inner and outer radius of the dielectric, respectively, *c* is the speed of light in vacuum equals 299,792,458 m/s, ${\rm E}$ is the electric field, and H is the magnetic field., the electric field within the tissue has a limited axial component, while the magnetic field is purely azimuth, so the wave equation can be expressed as:

5 $$\nabla \times \left( {\left( {\varepsilon_{r} - \frac{j\sigma }{{\omega \varepsilon_{o} }}} \right)^{ - 1} \nabla \times {\rm H}_{\varphi } } \right) - K_{o}^{2} \mu_{r} {\rm H}_{\varphi } = 0.$$

where $\varepsilon_{o}$ and $\varepsilon_{r}$ represent the dielectric constant in vacuum, and the relative dielectric constant of the tissue, respectively. Also, $\sigma$ is the tissue conductivity, and $\mu_{r}$ is the relative magnetic permeability of a medium. It is assumed that $\mu_{r} = 1$ for any used biological tissues. Furthermore, $K{}_{o}$ is the free-space wave number (1/m).

The thermal distribution within the different tissues is influenced by their thermophysical bioheat transfer properties [25]. One of the most standard models describing spatially/ temporally the temperature distribution within the tissues is the bio-heat equation, which was introduced by Pennes’ bioheat equation [30] as:

6 $$\rho c_{p} \frac{{\partial {\rm T}}}{\partial t} + \nabla \cdot ( - k_{th} \nabla {\rm T}) = Q + Q_{bio} ,$$

7 $$Q = \sigma_{eff} {\rm E}^{2} ,$$

8 $$Q_{bio} = \rho_{b} c_{p,b} \omega_{b} ({\rm T}_{b} - {\rm T}) + Q_{met} .$$

where $\rho$ is the tissue density(kg/m^3^), $c_{p}$ is the specific heat of the tissue which is the amount of the heat required to increase the tissue’s temperature by 1 °C per unit mass (J/(kg K)); ${\rm T}$ is the transient tissue’s temperature (K), and *t* is time. $k_{th}$ refers to the thermal conductivity that describes the ability of tissues to conduct heat (W/(m K)), which measures the ability of the tissues to conduct heat in relation to their ability to retain thermal energy. *Q* represents the external heat source produced by the microwave probe, $Q_{bio}$ indicates the heat generated in the body due to metabolism and blood. Also, $\rho_{b}$ is the blood density equals 1e^3^ (kg/m^3^), $c_{p,b}$ is the specific heat of the blood equals 3639 (J/(kgK)), $\omega_{b}$ is the blood perfusion rate equals 3.6e-3 (1/s), ${\rm T}_{b}$ is blood temperature equals 37 °C, and $Q_{met}$ is metabolic heat equals 0.

The term $\rho_{b} c_{p,b} \omega_{b} ({\rm T}_{b} - {\rm T})$ in the Pennes’ bioheat equation represents the heat exchange due to tissue blood perfusion, which plays a major role in regulating the temperature distribution within biological tissues during microwave ablation. The perfusion rate $\omega_{b}$ decreases with temperature as blood flow is reduced due to vascular coagulation above 50 °C.

The thermal damage or the degree of tissue injury is denoted by $\alpha$ and expressed by the Arrhenius equation [31] as:

9 $$\frac{\partial \alpha }{{\partial t}} = (1 - \alpha )^{n} {\rm A}e^{{{\raise0.7ex\hbox{${ - \Delta {\rm E}}$} \!\mathord{\left/ {\vphantom {{ - \Delta {\rm E}} {R{\rm T}_{abs} }}}\right.\kern-0pt} \!\lower0.7ex\hbox{${R{\rm T}_{abs} }$}}}} .$$

where A is a frequency factor depending on the used tissue, $\Delta {\rm E}$ is the activation energy depending on tissue, *n* is a polynomial order equal 1, *R* is the gas constant, and ${\rm T}_{abs}$ is the absolute temperature. The fraction of necrotic tissue $\theta_{d}$ is determined from the degree of the tissue injury according to the following equation [31]:

10 $$\theta_{d} = 1 - e^{ - \alpha } .$$

### Properties of the biological tissues

In this work, healthy and tumor tissues of different organs, namely liver, breast, kidney, lung, and were ablated using the proposed probes. The tumor was placed inside the healthy tissue to study the impact of the 3TSS and 10TSSS probes on both healthy and tumor tissues, as there are differences between the characteristics of healthy and the corresponding tumor tissues of the same organ as shown in Table 1.

**Table 1:** Properties of biological tissues of different organs [26].

Property	Variable	Unit	Value for different biological tissues							
			Healthy Liver	Liver tumor	Healthy Breast	Breast tumor	Healthy Kidney	Kidney tumor	Healthy Lung	Lung tumor
Heat capacity at constant pressure	Cp	J/(kg·K)	3540	3540	2960	2960	3980	3980	2560	2560
Density	Rho	kg/m^3^	1040	1160	980	1060	1050	1120	260	350
Thermal conductivity	k	W/(m·K	0.56	0.6	0.37	0.43	0.539	0.6	0.302	0.31
Frequency factor	A	1/s	7.39e^39^	7.39e^39^	7.39e^39^	7.39e^39^	7.39e^39^	7.39e^39^	7.39e^39^	7.39e^39^
Activation energy	dE	J/mol	2.577e^5^	2.577e^5^	2.577e^5^	2.577e^5^	2.577e^5^	2.577e^5^	2.577e^5^	2.577e^5^
Relative permittivity	$\varepsilon_{r}$	1	43.03	45	4.49	5	52.74	54	20.5	23
Relative permeability	$\mu_{r}$	1	1	1	1	1	1	1	1	1
Electrical conductivity	$\sigma$	S/m	1.69	2	0.59	1.1	2.43	3	2.5	3

The properties of the different human tissues differ as shown in Table 1. These values were used in the FEM simulation model to determine the effect of the proposed probes during the ablation process. Additionally, to find the required time for each probe to eradicate the whole tumor tissue regions, all tumors are assumed to have the same dimensions in the form of an ellipse with minor and major axis of 20 and 30 mm, respectively.

### Proposed thermal ablation probe structure

**Fig. 1.**
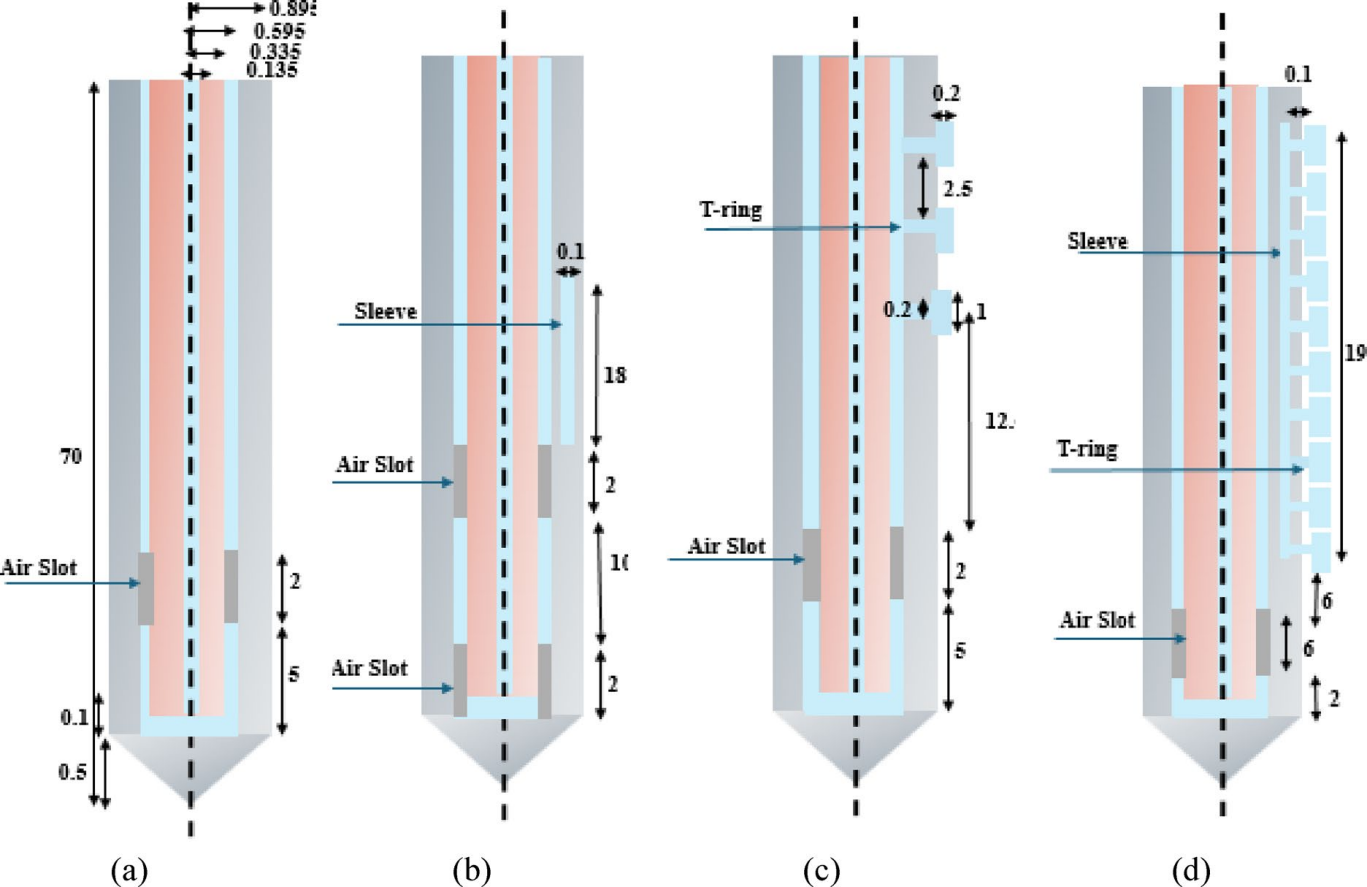
2D-axisymmetrical view of probes where, (**a**) traditional single air slot probe, (**b**) traditional double slot sleeved probe, (**c**) proposed 3TSS (single slot with 3T-rings) probe, and (**d**) proposed 10TSSS (single slot sleeved with 10T-rings).

In this work, the structure, and dimensions (in mm) of the two new probes: 3TSS probe, and 10TSSS probe. The new designs are based on the configuration of the traditional single air slot, and double slot sleeved probes [32] are illustrated in Fig. 1.

**Fig. 2:**
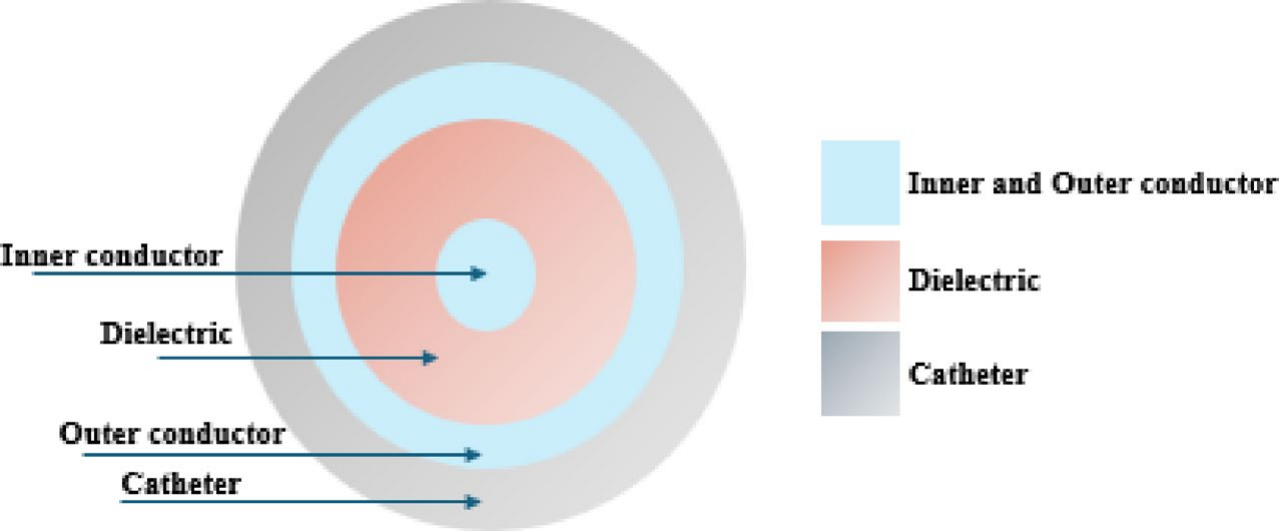
The cross-section view of the probes, where each color represents a different material: conductor , dielectric , and catheter .

Figure 1 shows the arrangement of each probe, which consists of four materials, namely conductor, dielectric, catheter, and air. The first traditional probe has only one air slot at a height of 5.5 mm from the probe tip, while the second traditional probe has two air slots at different heights 0.5 mm and 12.5 mm and a sleeve as well. On the other hand, the structures of the proposed two new probes are: (i) the 3TSS contains one air slot and three T-rings that protrude 0.2 mm outside the probe body based on trial-and-error experiments, where their location and the distances between them control the shape of the thermal emission and electromagnetic waves generated by the probe, and (ii) the proposed 10TSSS probe contains one air slot and a 19 mm long sleeve with ten T-rings These values are after trying many values to get the best performance of each probe. In addition, the cross-sections of the probes are the same as shown in Fig. 2.

**Fig. 3:**
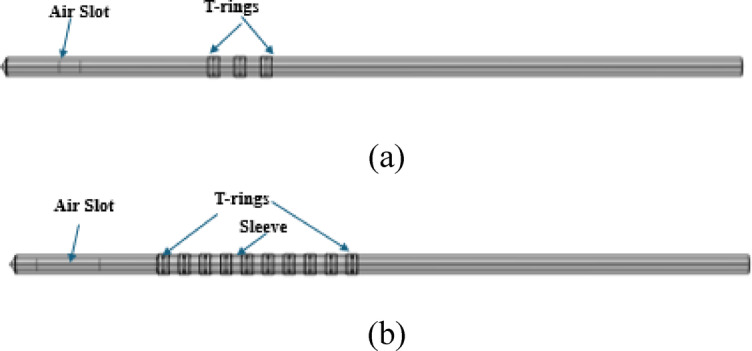
The 3D view of proposed probes: (**a**) single slot with 3T-rings probe, and (**b**) single slot sleeved with 10 T-rings probe.

In Figs. 1 and 2, the probes’ body consists of four different materials as shown in different colors: light gray, brown, and dark gray for the cathode, conductor, dielectric, and air slot, respectively. The three-dimensional views of the two proposed probes presenting each at length of 70 mm and diameter of 2.19 mm are demonstrated in Fig. 3.

The electrical properties of the materials used in the probes’ design are listed in Table 2 [33]. Each material has distinctive properties that affect the conduction of heat and electromagnetic waves and their emission from the probe.

**Table 2:** Electric properties of the materials used in the probes [33].

Property	Variable	Unit	Value for different materials		
			Catheter	Dielectric	Air Slot
Relative permittivity	$\varepsilon_{r}$	1	2.6	2.03	1
Relative permeability	$\mu_{r}$	1	1	1	1
Electrical conductivity	$\sigma$	S/m	0	0	0

The dielectric layer surrounding the inner conductor was modeled as Polytetrafluoroethylene (PTFE, Teflon). This material is widely used in microwave ablation probes due to its low relative permittivity (εr = 2.03) and high temperature tolerance of up to approximately 260 °C. To evaluate the thermal heating/ablation abilities of the proposed probes, the FEM was used for modeling the ablation process by simulating both the probes and the healthy/tumor tissues of different organs being ablated. The simulation was performed using healthy tissue with dimensions of 90 and 40 mm, and inside it was tumor tissue in the form of an ellipse with radii dimensions of 15 and 10 mm. Simulation facilitates the acquisition of results that more accurately reflect reality by employing properties that closely resemble those of materials utilized in real experiments. Additionally, it allows significant reduction in costs and enables an unlimited number of experiments to be conducted. Thereby, it optimizes the results while minimizing the resources consumption to adapt with the real-world clinical practices. The arrangement of mesh elements within the finite mathematical model illustrates the areas where calculations are most prevalent, particularly in proximity to the probe, to achieve results that closely align with actual experimental outcomes [34]. The number and dimensions of the mesh units affect the accuracy and time of the simulation, as dimensions must be chosen that are consistent with the dimensions of the different parts of the simulation model. The simulation model consists of three parts: healthy tissue, tumor tissue, and the probe.

**Fig. 4:**
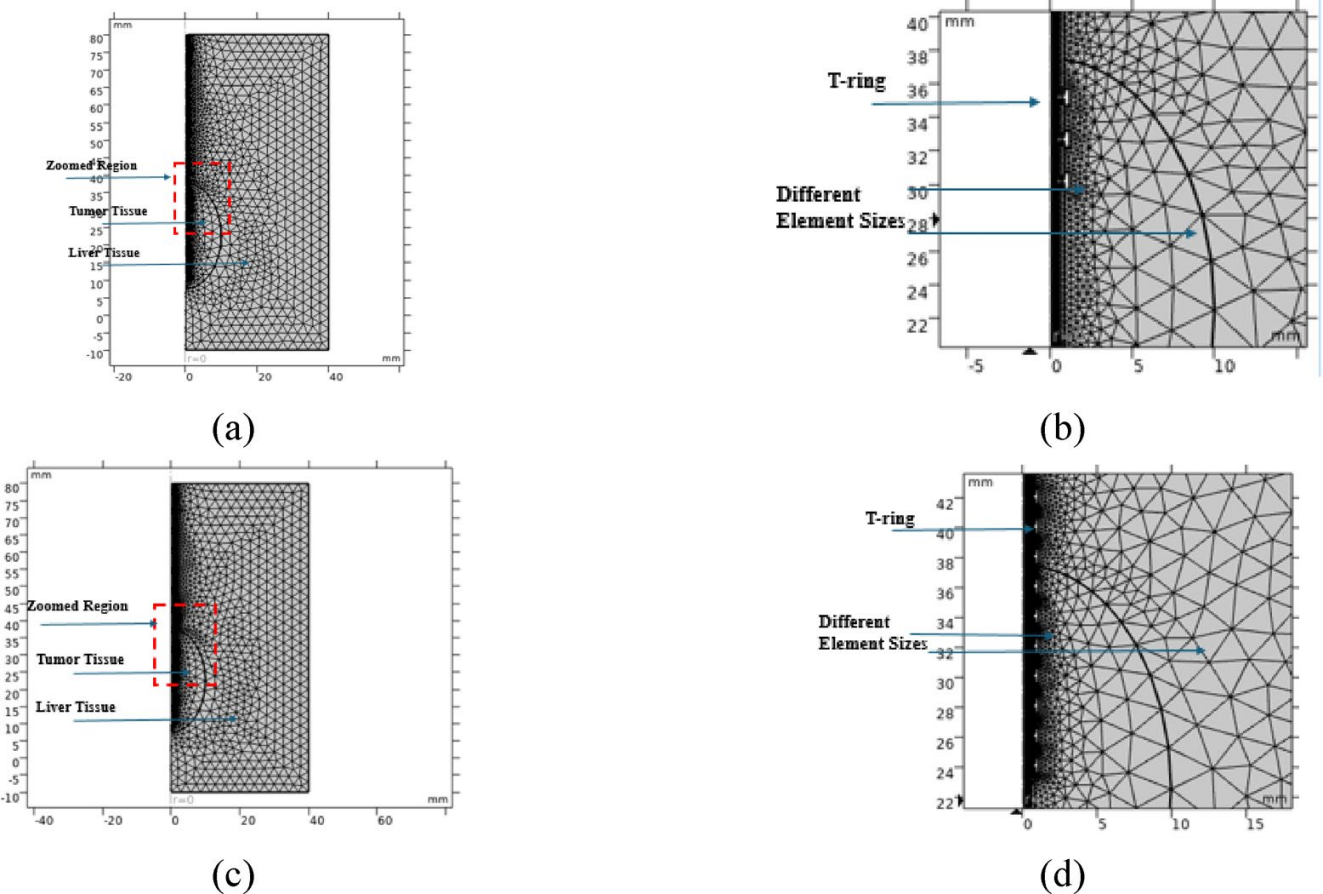
The finite element model mesh structure: (**a**, **b**) complete structure and zoomed area, respectively, using the proposed 3TSS probe, and (**c**, **d**) complete structure and zoomed area, respectively, using the proposed 10TSSS.

In this work, the finite element model incorporates smaller elements specifically in the locality of the probe and at the interfaces between different tissues, while in regions where the tissue’s area is more extensive, larger finite elements are employed. The simulation was configured with the following parameters: 3 mm maximum allowable size of the element within the tissues, 0.024 mm minimum element’s size, 1.3 maximum growth rate of the element, and 0.3 curvature factor is established at 0.3. These values were used to provide accurate mathematical results, whereas the size of a single element increases, and the calculations become faster. However, more than one change can occur within it, and thus the results are less accurate and far from reality. The probe’s design incorporates small components, characterized by the diminutive size and numerous mesh units, with the largest dimension in the probe area 0.15 mm as shown in Fig. 4.

### Evaluation metrics

To evaluate the proposed probes, different valuation metrics were measured, including the dissipated power density in W/m3(also known as SAR, specific absorption rate), which is the dissipated power divided by the volume and. Its numerical value can be formulated as an expression of the power dissipated from the probe and thus transferred from the probe to the surrounding tissues, where

11 $$\Gamma = \frac{{P_{reflected} }}{{P_{incident} }},$$

12 $$RL = - 20\log (\left| \Gamma \right|),$$

13 $$S_{11} = - RL = 20\log (\left| \Gamma \right|).$$

where S11 represents the amount of power reflected from the probe. The reflection coefficient (gamma or return loss) [8, 35], where $\Gamma$ is a ratio representing the reflection coefficient and $\Gamma < 1,P_{reflected}$ is the reflected power, $P_{incident}$ is the total incident power, *RL* is the return loss and always have a positive value, and $S_{11}$ is the *s* parameter in dB which is the negative of the $RL$ and is a negative dB value. The measurement of S11 provides a reliable indicator of the probe’s effectiveness as S11 is equal to 0 dB and signifies that all power is being reflected by the probe, resulting in no radiation. Conversely, negative S11 values indicate a reduction in the power reflected from the probe, with most of the power being radiated, which aligns with the intended function of the probe.

## Experimental results

### Total power dissipation density

**Fig. 5:**
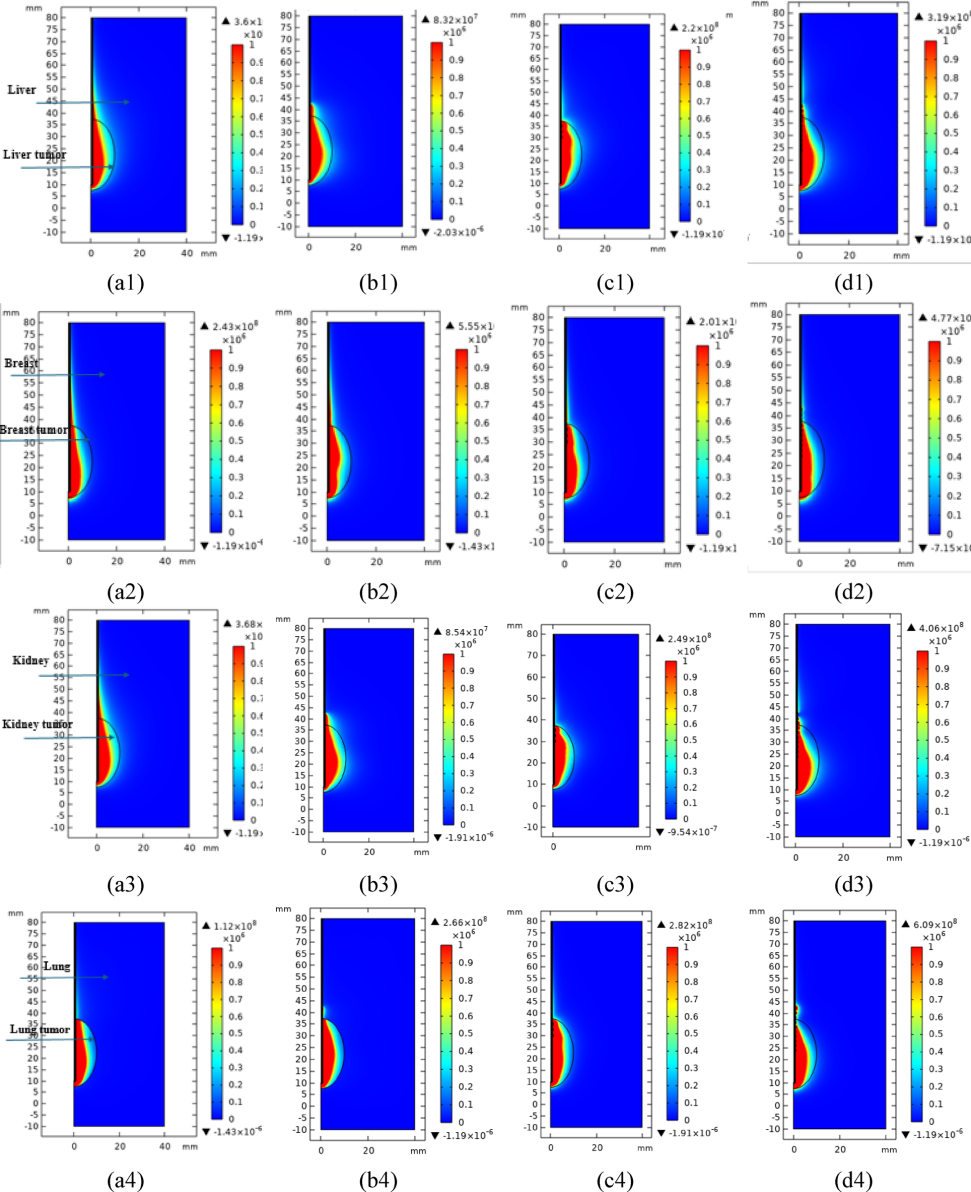
Total power dissipation density in W/m^3^ in different organs in the four tissues including the tumors, namely liver, breast, kidney and lung, as represented in the first left column to the last 4th column, respectively, where (a1-4) single air slot probe, (b1-4) double slot sleeved probe, (c1-4) single slot with 3 T-rings probe, and (d1-4) 10TSSS probe and the color bar’s legends in W/m^3^.

To examine the correlation between the temperature and the necessary exposure duration, it is essential to consider the configuration of the generated radiation probe as well as the requisite temperature for complete ablation, which differs based on the type of probe, and the frequency used for producing microwave energy. The power used is 10 Watts for each probe at 2.45 GHz microwave frequency. In addition, the dissipated power density from the probe which transferred to the surrounding tissues is illustrated in Fig. 5. It expresses the probe’s consumption of the power it was fed. All probes were fed with the same power of 10 watts for the same period of 10 min. However, the form of the power loss and its value differs depending on the probe and its internal composition.

In Fig. 5, the red color refers to the highest value of the power dissipation and thus the conversion of the lost power into thermal energy in the tissues surrounding the probe. Since the tissues that are ablated, whether tumor tissue or healthy tissue, the form of the power dissipated by each probe fundamentally affects the form of the ablated tissues as well as the form of the thermal emission around the probe. This occurs depending on the relationship between the time and the heat to which the tissue is exposed.

The shape of the power dissipated between the four probes in the different tissue in the presence of an elliptical tumor with dimensions of semi-major axis 15 mm and semi-minor axis 10 mm around each probe. Nevertheless, the single air slot probe has a backward tail along the probe upwards, which will cause tissue ablation around the probe upwards. Also, the double slot sleeved probe has a backward tail, which is slightly less than the backward tail using the single air slot probe, nonetheless there is still a slight power loss upwards along the probe. It is also noticeable that the shape of the dissipated power changes according to the probe used, and the type of tissue being ablated. The 3TSS, and 10TSSS probes give more concentration of the dissipated power in the tumor tissue. The results in Fig. 5 proved that using the proposed 3TSS, and 10TSSS probes presented most of the concentrated power loss is confined between the probe tip and the last T-ring along the probe. This in turn can control the shape of the dissipated power by the location of the T-rings, and they were moved to confine the power dissipation or energy emission to the tumor tissue region only.

### Ablated tissue

**Fig. 6:**
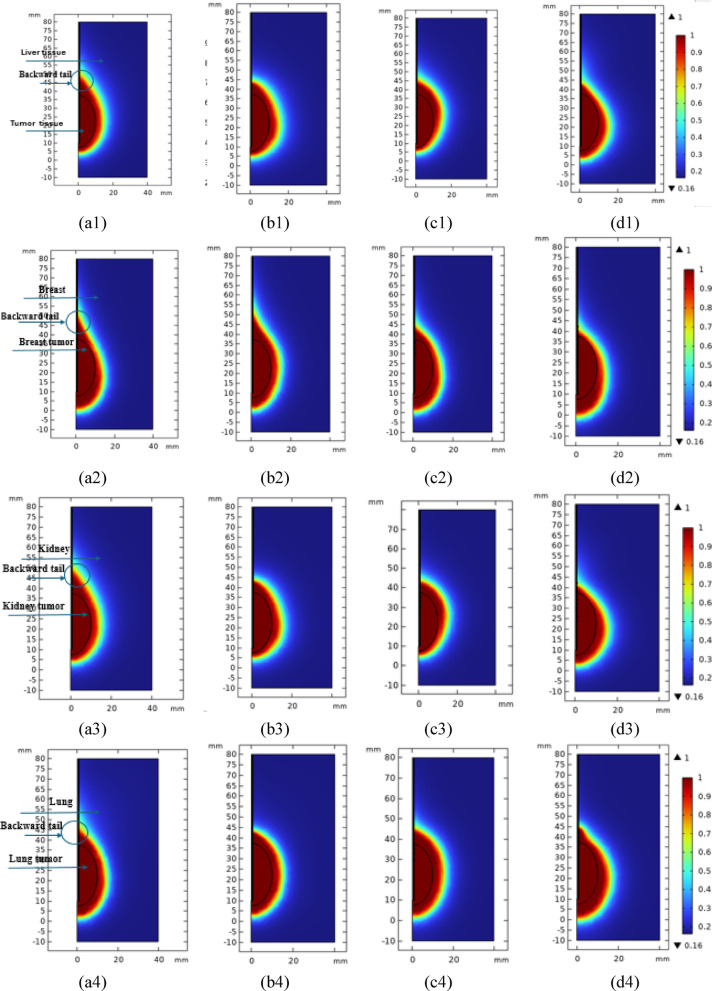
The 2D-axisemitrical ablated tissue for the four probes in the four tissues, namely liver, breast, kidney and lung, as represented in the first left column to the last 4th column, respectively, where (a1-4) single air slot probe, (b1-4) double slot sleeved probe, (c1-4) single slot with 3 T-rings probe, and (d1-4) 10TSSS probe and the color bar’s legends show the degree of damage.

To reduce the ablation in healthy tissues as much as possible, different four probes were used to determine the best desired shape for tumor tissue ablation from different biological tissues as shown in Fig. 6.

The shape of the ablated tissue using each of the four probes in the different tissues exhibits an oval tumor region around each probe. The single air slot probe produces a small upward posterior ablation due to a residual tail of power dissipated along the probe length. Also, the double slot sleeved probe demonstrates a reduced posterior energy tail, though a slight upward ablation along the probe length remains observable. The distribution of the dissipated power strongly influences the overall ablation shape. The proposed 3TSS and 10TSSS probes provide enhanced control over the power dissipation between the probe tip and the final T-loop along the probe. This effectively confined the ablation to the target tumor tissue at the probe’s distal end. This design minimizes unwanted posterior ablation without requiring additional cooling mechanisms, thereby simplifying the physician’s procedure. Although all probes achieved complete tumor ablation, the proposed designs (3TSS and 10TSSS) exhibited superior performance by confining the ablation to the target region and minimizing unnecessary heating of surrounding healthy tissue.

In addition, backward heating along the probe axis and feed line is a well-known problem in microwave ablation. Previous studies have shown that water-cooling the probes can significantly reduce the backward heating and improve the homogeneity of ablation zones [36, 37]. Similarly, gas-cooling/cryogenic cooling methods have been shown to effectively limit axis heating while maintaining the desired ablation profile [38]. However, these cooling methods have several disadvantages, namely water-cooling typically requires additional fluid handling, larger probe diameters to accommodate cooling channels, the risk of leakage or contamination, and potential sterilization complexity. Cryogenic or gas-cooling often involves more complex and expensive hardware and is potentially impractical in resource-limited settings.

The shape of the ablated tissues differs in healthy tissues compared to the tumor tissues based on the different shapes of the power dissipation or the microwave energy emission from each probe. The ablated regions of healthy tissue should preferably be around the tumor as a marginal region to reduce the chances of the tumor returning. The 10TSSS probe is considered the best for liver tissue’s ablation, while the 3TSS probe is the best for breast, kidney, and lung tissue’s ablation. Table 3 shows the friction of damage in the tissues after 10 min of using 10W microwave power with different probe structures.

**Table 3:** Ratio of damage in tissues after 10 min of using 10W microwave power using the proposed two proposed compared to using the traditional ones.

Probe type	Ratio of damage							
	Liver Tumor	Healthy Liver	Breast Tumor	Healthy Breast	Kidney Tumor	Healthy Kidney	Lung Tumor	Healthy Lung
Single Air Slot	1	0.21153	1	0.16181	1	0.16191	1	0.16238
Double Slot Sleeved	1	0.16200	1	0.16183	1	0.16178	1	0.16300
Single Slot with 3 T-rings	1	0.16204	1	0.16177	1	0.16184	1	0.16101
Single Slot Sleeved with 10 T-rings	1	0.16218	1	0.16085	1	0.16186	1	0.16151

It is required that the ablated part be completely around the tumor to prevent tumor cells from regenerating or spreading after the treatment procedures are completed [39]. Accordingly, using the proposed probes, with all cases, the tumor was completely ablated, as well as a marginal percentage of the surrounding healthy tissue around the tumor. It is noted in Table 3 that the percentages of healthy tissue ablated are close, but when looking at Fig. 6, which shows the ablated tissues, their shape differs depending on the type of both the tissue and the probe used. It is obvious that the ablation of healthy tissue around the entire tumor is best in the 10TSSS probe in liver and lung tissues, and in the 3TSS probe in all tissues.

The 3TSS and 10TSSS probes demonstrate superior ablation performance due to their optimized design, which allows better control of the electromagnetic field distribution. The T-shaped slots help to focus the energy on the tumor region, reducing backward energy loss and creating a more uniform ablation zone around the tumor. Although the total percentage of the ablated healthy tissue appears similar among all probe types. The 3TSS and 10TSSS designs achieve more desirable ablation shape—surrounding the tumor with an appropriate margin of healthy tissue to ensure complete tumor removal while minimizing the damaged tissue to adjacent areas. As illustrated in Fig. 6, the ablation regions produced by the 3TSS and 10TSSS probes are clearly demonstrate this effect. A more uniform and symmetric distribution of the ablated tissue is shown surrounding the tumor. This visual evidence supports the data in Table 3 and confirms that the improved probe designs achieve more controlled and clinically effective ablation patterns compared with the conventional single slot and double slots sleeved probes. The ablation pattern of the more effective designs should be extended slightly into the surrounding healthy tissue. Such extra area is forming a circumferential/safety margin around the tumor that corresponds to the clinically required safety zone for complete tumor eradication.

### Isothermal contours

**Fig. 7:**
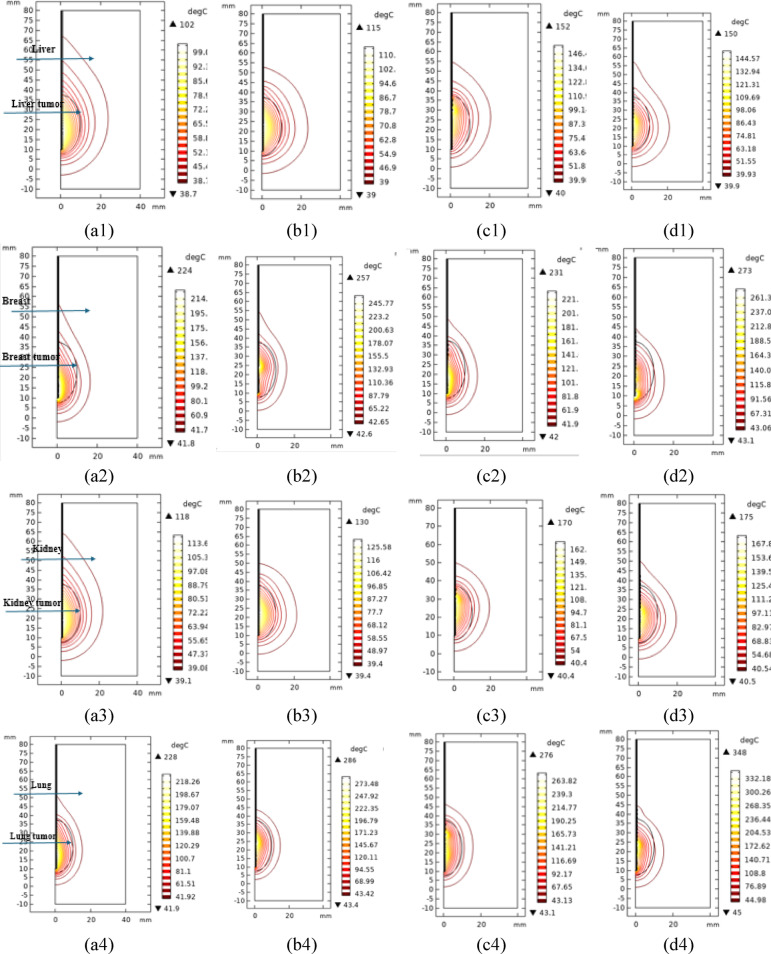
The 2D isotherm contours in the four tissues, namely liver, breast, kidney and lung, as represented in the first left column to the last 4th column, respectively, for the four probes: (**a**) single air slot probe, (**b**) double slot sleeved probe, (**c**) 3TSS probe, and (**d**) 10TSSS probe in the four tissues liver, breast, kidney and lung respectively, and the color legends shows the temperature in °C.

The ablated tissue depends on the heat generated by each probe and the shape of the isothermal contours around it as shown in Fig. 7.

The temperatures differ in the isotherm contours according to the type of probe used and the tissue. The highest temperature values after 10 min with the single air slot, double slot sleeved, 3TSS, and 10TSSS probes were 102 °C, 115 °C, 152 °C and 150 °C, respectively for the liver tissues. For the breast tissues, using the same probes, the temperatures become 224 °C, 257 °C, 231 °C and 273 °C, respectively. For the kidney tissues, the temperatures become 118 °C, 130 °C, 170 °C and 175 °C, respectively., For the lung tissues, the temperatures become 228 °C, 286 °C, 276 °C and 348 °C, respectively. Therefore, the generated temperature values are higher using the two proposed probes, leading to better consumption of the used power. It is also noticeable that the produced temperature by the single air slot probe increases along the length of the probe backward, leading to the eradication of healthy tissues around the probe. Using the double slot sleeved and 3TSS probes, the shape of the isotherm contours is spherical around the beginning of the probe. This shape is considered the preferred one to eradicate the tumor around the beginning of the probe only. The 10TSSS probe does exhibit backward heat; however, this effect is less pronounced compared to the produced one using the single air slot probe. Consequently, there is no substantial destruction of healthy tissue surrounding the probe, as illustrated in Fig. 6.

### Reflection coefficient

**Fig. 8:**
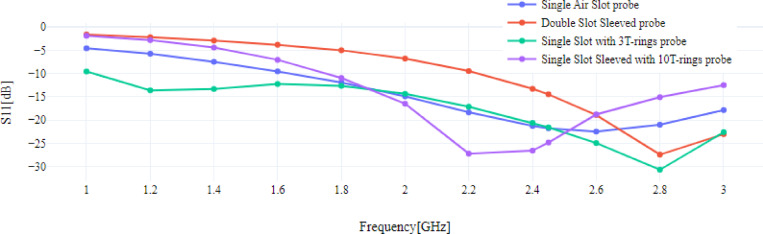
Reflection coefficient of the MW probes.

Moreover, Fig. 8 shows the reflection coefficient (S11) curve for each probe at frequency range 1–3 GHz.

The shape of the S11 curve for each probe varies according to the used frequency, thus, in this study we used 2.45 GHz [40]. The best value is the lowest value, as the lower the reflection coefficient value means that more power is consumed and transferred better and with less waste from the probe used to the surrounding tissues. The best result was achieved for S11 using the proposed 10TSSS.At the microwave frequency of 2.45 GHz, the values of S11 for the single air slot probe, double slot sleeved probe, 3TSS, and 10TSSS are -21.752 dB, -14.455 dB, -21.553 dB, and -24.816 dB, respectively.

### Mass power dissipation

**Fig. 9:**
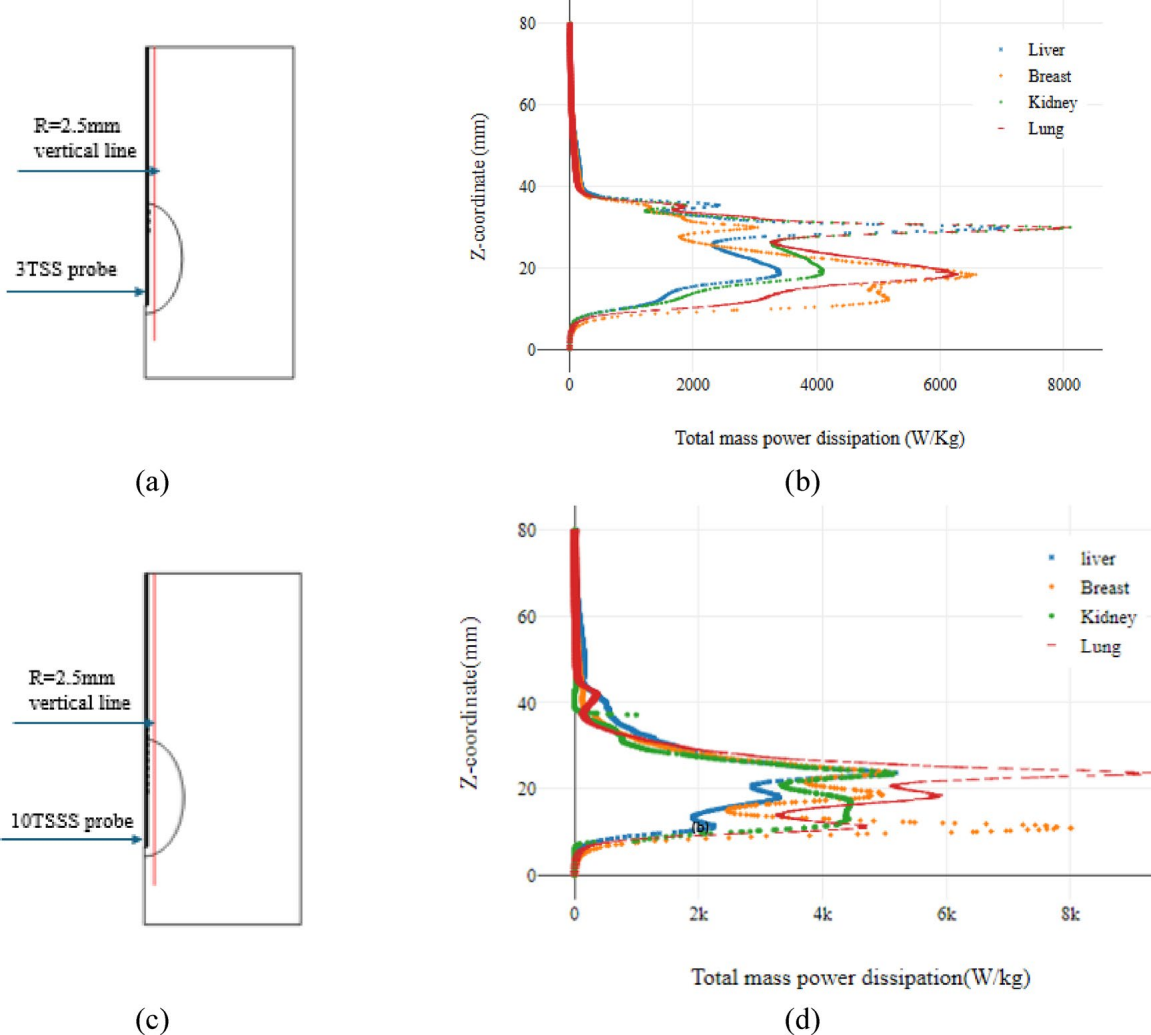
The total mass power dissipation along the R = 2.5 mm vertical line using the proposed 3TSS, and 10TSSS probes, where (**a**, **c**) 2D cut line, and (b,d) the total mass power dissipation for different organs in (W/kg) for 3TSS, and 10TSSS probes respectively.

Since the total dissipated power is the energy consumed in the tissue model [41] which was consumed to heat the tissues surrounding the probe. Figure 9 shows its value using the proposed 3TSS, and 10TSSS probes to study the difference in its effect on different tissues of the liver, breast, kidney and lung.

The value of the total dissipated power was calculated along a vertical line at a distance 2.5 mm from the center of the sample as shown in Fig. 9a, c, and the values are shown in Fig. 9 (b, d) which points out the difference in the power values along the line at which the calculation is done. Using the same probe, the value of the total dissipated power at the same point differs according to the type of tissue. The highest values of the dissipated power occur with the lung tissue ablation due to the tissue properties which lead to the highest temperatures. The difference in the shape of the curves shows the difference in the shape of the dissipated power radiation for each tissue as shown in Fig. 5.

### Fraction of damage

**Fig. 10:**
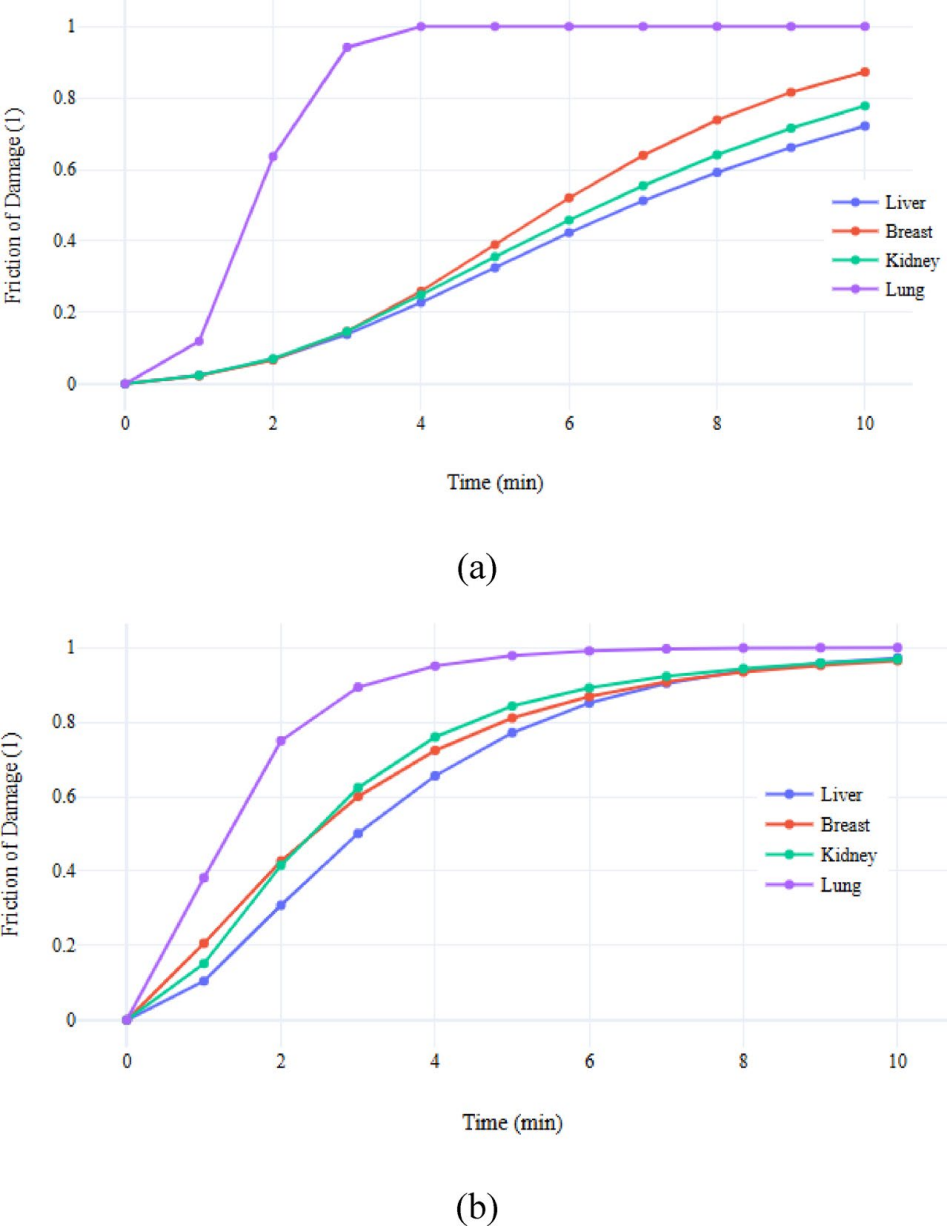
Fraction of damage for (**a**) 3TSS probe, and (**b**) 10TSSS probe in different organs using 5W.

The dissipated power varies depending on the type of tissue under consideration. Therefore, to conduct an accurate study of any probe, it should be tested on the same type of tissue in which it will be used. The greater the amount of power dissipated within the tissue, the more rapidly it will undergo ablation, as increased power consumption leads to a quicker rise in temperature. This ultimately results in the ablation of the tissue. Since the dissipated power value in the lung tissue is the largest, the lung tissue is ablated in a shorter time compared to the other tissue types as shown in Fig. 10.

Using the 3TSS, and 10TSSS probes for 10 min and 5 watts power, a difference in the friction of damage of different tissues is shown in Fig. 10. The tumor located in the lung tissue was successfully ablated approximately 4 and 5 min after the initiation of the ablation process for the 3TSS, and 10TSSS probes, respectively. In contrast, the ablation of the liver, kidney, and breast tissues required a longer duration to achieve complete tumor removal. It is important to highlight that as the power utilized in the ablation process increases, the duration needed to ablate the tumor across various tissues diminishes. A power level of 5 watts was employed to demonstrate the variation in the speed of tumor ablation in lung tissue. This is attributed to the significant increase in the amount of power dissipated within the lung tissue in comparison to other types of tissue. A power level of 5 W was selected to emphasize the differences in heat distribution among the various tissue models and probe types. At higher powers (e.g., 10 W), all configurations reached complete ablation rapidly, producing similar outcomes and masking the distinctions between tissue responses.

## Discussion

**Fig. 11:**
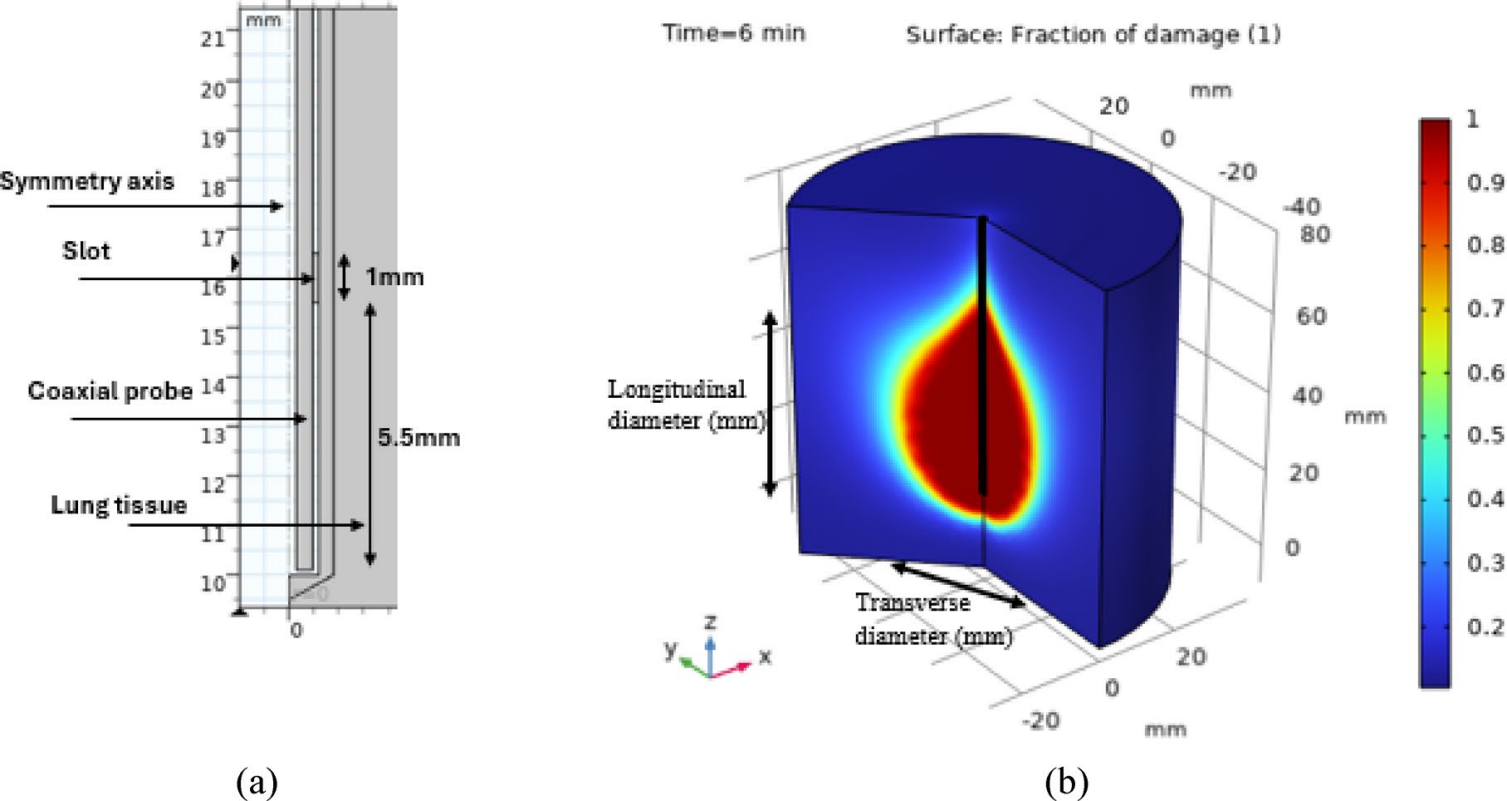
The simulation for the proposed probe in [42] for comparison, where (**a**) single air slot probe, (**b**) the ablated porcine lung tissue.

This work used the FEM to simulate the ablation of various healthy and tumor tissues found within distinct tissue types across different organs. The simulation framework is grounded in a numerical analysis of a finite number of elements within the model, with dimensions that are adjusted according to the specific design of the model. To assess the accuracy of our study in relation to real-world conditions, we conducted a comparison with a simulation model that was developed under identical parameters, including the working environment, the tissues utilized, the type of probe, power settings, duration, and all operational conditions outlined in the research that performed a practical experiment on porcine lung tissues. We compared the results from the simulation model with the results of the practical/clinical experiment to show the extent of convergence or difference between them. Using a single air slot probe on ex-vivo porcine lungs at a frequency of 2.45 GHz with a power of 30, 40 and 50W is found in the paper [42]. Our results are compared with the results of this practical experiment using the same probe and the same tissue properties. Thus, the same probe used in [42] was designed with the same dimensions as shown in Fig. 11.

For comparison, the single air slot probe was used at 30, 40, 50 W for 6 min to ablate non-tumor porcine lung tissue as performed by Geo et al. [42]. Table 4 shows the differences between the results of our study and the practical experiment in [42].

**Table 4:** A comparative study at 2.45 GHz after 6 min of microwave ablation using 30, 40, and 50W compared to [42].

Input power = 30W		
	Practical Experimental setting by Geo et al. [42]	Our simulation data
Transverse diameter of ablated tissue (mm)	29.5 ± 4.3	29.6 ± 1.2
Longitudinal diameter of ablated tissue (mm)	41.1 ± 3.8	48.5 ± 1.6
Input power = 40W		
Transverse diameter of ablated tissue (mm)	44.0 ± 2.3	38.5 ± 2.2
Longitudinal diameter of ablated tissue (mm)	55.7 ± 4.3	55.0 ± 1.2
Input power = 50W		
Transverse diameter of ablated tissue (mm)	48.9 ± 3.8	41.2 ± 3.7
Longitudinal diameter of ablated tissue (mm)	66.3 ± 4.9	62.4 ± 4.5

The results presented in Table 4 indicate a notable correlation between the outcomes of the simulation model and those obtained from practical experiments. This proves that the simulation model’s results can be considered dependable, assuming that identical values are applied to the materials and tissues utilized in the experiments. Consequently, this approach contributes to a reduction in both study duration and expenses.

In the future work, it is recommended to fabricate the proposed probe designs and conduct experimental verification to confirm the effectiveness of the proposed probes and support the simulation results presented in this study. Moreover, the proposed 3TSS and 10TSSS antennas were designed considering realistic geometries, commercially available coaxial materials, and standard manufacturing techniques. Therefore, their fabrication is feasible and will be considered in future work to experimentally validate the simulated results. Although the present work is based on numerical simulation, it provides valuable insight into the electromagnetic and thermal performance of the proposed probe designs. These findings establish a foundation for future experimental validation, where the probes can be fabricated using standard coaxial materials and tested under realistic clinical conditions.

The simulations presented here characterize each probe design independently to provide clear baseline comparisons of power dissipation, isothermal contours, and ablation shape. We acknowledge that multi-antenna treatment strategies are commonly used clinically for large tumors and that antenna phase relationships can strongly affect the resulting thermal pattern. The designs of the proposed probes are optimized for small to moderate tumor volumes, where uniform heating and complete ablation can be achieved with a single insertion. For larger tumors, increasing the input power or ablation time can extend the ablated region; however, this may lead to unwanted thermal injury to adjacent healthy tissues. In future work, combining multiple single probe insertions or employing phased-array configurations could provide a practical approach to achieve complete ablation of large tumors.

The present study applied power levels of 5–10 W to ensure safe and comparable evaluation across antenna designs for tumors of 20 × 30 mm. On the other side, the clinical studies indicate that higher powers (e.g., 40–150 W) are used to treat large lesions in short ablation time and greater volume of necrosis [43]. In the future work, it is recommended to apply higher power settings to assess how the proposed 3TSS and 10TSSS probes perform in the clinical realistic scenarios, particularly in terms of reduced ablation time and full tumor coverage.

Once the tissues temperature exceeds 100 °C, water content starts to vaporize, producing steam and vapor bubbles. This process changes the dielectric properties of the tissue and may reduce microwave energy absorption. Thereby limiting further temperature increase and affecting the ablation zone’s shape [44]. During MWA, tissue shrinkage may occur as a result of water evaporation and thermal contraction, leading to a reduction in the apparent size of the ablated region compared to the initial target volume. This phenomenon should be considered when correlating the simulation results with the experimental/ clinical findings [45]. Generally, the present study focused on the ablation of relatively small tumor volumes. In future work, larger tumor sizes will be investigated, which will require higher input powers up to 150 W. Moreover, the performance of probe arrays and the implementation of cooling mechanisms will be explored to ensure patient safety during the ablation procedure.

## Conclusions

An effort was undertaken to implement various types of microwave ablation probes. Two specific types demonstrated superior performance compared to the alternatives being evaluated, particularly regarding the power consumption utilized and the energy dissipated within the tissues undergoing ablation. The comparison was conducted between the single air slot probe, double slot sleeved probe, single slot with 3 T-rings probe, and single slot sleeved with 10 T-rings probe in terms of their internal structure and the shape of the power dissipated by each of them. Consequently, the shape of the tissue ablated as a result of each probe ablation process, which depends on the shape of the isotherm contours.

It was found that the values of the dissipated power of the proposed 3TSS, and 10TSSS probes are higher, which led to the heat values resulting from the probes being higher, as appeared in the isotherm contours resulting from each probe which were 152 °C and 150 °C, respectively for the liver tissues compared to 102 °C, 115 °C, of the two traditional probes for the same tissue type. We attributed this to the values of S11 or the reflection coefficient of the two probes which were lower compared to the other two probes. This means that more power has been radiated from the developed probes, which means that they are better than the other two probes in power consumption.

A comparison was carried out for four types of human tissues, namely the liver, breast, kidney, and lung. Each tissue has a tumor inside it in the form of an ellipse. The 3TSS, and 10TSSS probes were chosen to make this comparison in terms of the shape of the dissipated power along a vertical line parallel to the probe and how the values of the dissipated power differ depending on the type of tissue. Thus, the speed of ablation of each tissue differs. The 3TSS, and 10TSSS probes presented were the best in the form of the ablated part around the tumor from all directions to ensure that the tumor would not return after the end of treatment procedure. The 10TSSS probe gave the highest temperature compared to the rest of the probes, as the maximum temperature resulting when ablating the lung tissues was 348 °C compared to 228, 268 and 276 °C for the single air slot, double slot sleeved and 3TSS probes, respectively. The 3TSS probe was the fastest in ablating the lung tissues as it takes only 4 min to ablate the entire tumor using power of 5W, while the 10TSSS probe takes 6 min to ablate the tumor under the same conditions. From the results obtained, the use of these proposed probes in clinical trials will achieve complete tumor eradication in a shorter time than traditional probes.

## Data Availability

No clinical data is associated with this article. The COMSOL model files used to generate the results presented in this paper are available from the corresponding author upon reasonable request.
