# Publisher Correction: Novel T-ring-based probes for thermal ablation of tumors in different organs

**DOI:** 10.1038/s41598-026-62032-1

**Published:** 2026-07-16

**Authors:** Menna Asran, Mahmoud Farouk Selim, Amira S. Ashour

**Affiliations:** 1 Department of Electronics and Electrical Communications Engineering, Faculty of Engineering, Tanta University, Tanta, Egypt; 2 Faculty of Medicine, Tanta University, Tanta, Egypt

Correction to: *Scientific Reports* 10.1038/s41598-025-28192-2, published online 05 December 2025

The original version of this Article contained an error in Reference 1.

As a result, in the Reference list:

“1. Kurgan, E. & Gas, P. “Estimation of temperature distribution inside tissues in external RF hyperthermia. Blood **1060**(3639), 310–315 (2010).”

now reads:

“1. Kurgan, E. & Gas, P. Estimation of Temperature Distribution Inside Tissues in External RF Hyperthermia. *Przeglad Elektrotechniczny* **86**(1), 100–102 (2010).”

The original Article has been corrected.

